# Supplementary material for: Relationship between acute glucose variability and cognitive decline in type 2 diabetes: A systematic review and meta-analysis
Source: PLoS One. 2023 Sep 1;18(9):e0289782. doi: 10.1371/journal.pone.0289782 (PMC10473499; doi:10.1371/journal.pone.0289782)
Supplement: S1 File — (DOC) [file pone.0289782.s002.doc]

**NEWCASTLE - OTTAWA QUALITY ASSESSMENT SCALE**

**CASE CONTROL STUDIES**

Note: A study can be awarded a maximum of one star for each numbered item within the Selection and Exposure categories. A maximum of two stars can be given for Comparability.

1.Glucose Fluctuations Are Linked to Disrupted Brain Functional Architecture and Cognitive Impairment

**Selection**

1) Is the case definition adequate?

a) yes, with independent validation **🟑**

b) yes, eg record linkage or based on self reports

c) no description

2) Representativeness of the cases

a) consecutive or obviously representative series of cases **🟑**

b) potential for selection biases or not stated

3) Selection of Controls

a) community controls **🟑**

b) hospital controls

c) no description

4) Definition of Controls

a) no history of disease (endpoint)

b) no description of source**🟑**

**Comparability**

1) Comparability of cases and controls on the basis of the design or analysis

a) study controls for _______________ (Select the most important factor.) **🟑**

b) study controls for any additional factor **🟑** (This criteria could be modified to indicate specific control for a second important factor.)

**Exposure**

1) Ascertainment of exposure

a) secure record (eg surgical records) **🟑**

b) structured interview where blind to case/control status **🟑**

c) interview not blinded to case/control status

d) written self report or medical record only

e) no description

2) Same method of ascertainment for cases and controls

a) yes **🟑**

b) no

3) Non-Response rate

a) same rate for both groups **🟑**

b) non respondents described

c) rate different and no designation

2.Effects of glycemic variability and hyperglycemia in acute ischemic stroke on post-stroke cognitive impairments.

**Selection**

1) Is the case definition adequate?

a) yes, with independent validation **🟑**

b) yes, eg record linkage or based on self reports

c) no description

2) Representativeness of the cases

a) consecutive or obviously representative series of cases**🟑** 

b) potential for selection biases or not stated

3) Selection of Controls

a) community controls **🟑**

b) hospital controls

c) no description

4) Definition of Controls

a) no history of disease (endpoint) **🟑**

b) no description of source

Comparability

1) Comparability of cases and controls on the basis of the design or analysis

a) study controls for _______________ (Select the most important factor.) **🟑** 

b) study controls for any additional factor  (This criteria could be modified to indicate specific control for a second important factor.)**🟑**

Exposure

1) Ascertainment of exposure

a) secure record (eg surgical records) **🟑**

b) structured interview where blind to case/control status**🟑** 

c) interview not blinded to case/control status

d) written self report or medical record only

e) no description

2) Same method of ascertainment for cases and controls

a) yes **🟑**

b) no

3) Non-Response rate

a) same rate for both groups 

b) non respondents described**🟑**

c) rate different and no designation

1. Relationship between peripheral neuropathy and cognitive impairment in elderly patients with type 2 diabetes mellitus

**Selection**

1) Is the case definition adequate?

a) yes, with independent validation**🟑** 

b) yes, eg record linkage or based on self reports

c) no description

2) Representativeness of the cases

a) consecutive or obviously representative series of cases **🟑** 

b) potential for selection biases or not stated

3) Selection of Controls

a) community controls 

b) hospital controls**🟑**

c) no description

4) Definition of Controls

a) no history of disease (endpoint) 

b) no description of source

Comparability

1) Comparability of cases and controls on the basis of the design or analysis

a) study controls for _______________ (Select the most important factor.) **🟑** 

b) study controls for any additional factor  (This criteria could be modified to indicate specific control for a second important factor.)**🟑**

Exposure

1) Ascertainment of exposure

a) secure record (eg surgical records) **🟑**

b) structured interview where blind to case/control status **🟑**

c) interview not blinded to case/control status

d) written self report or medical record only

e) no description

2) Same method of ascertainment for cases and controls

a) yes**🟑** 

b) no

3) Non-Response rate

a) same rate for both groups**🟑** 

b) non respondents described

c) rate different and no designation

1. The relationship between glucose excursion and cognitive function in aged type 2 diabetes patients

**Selection**

1) Is the case definition adequate?

a) yes, with independent validation **🟑**

b) yes, eg record linkage or based on self reports

c) no description

2) Representativeness of the cases

a) consecutive or obviously representative series of cases 

b) potential for selection biases or not stated**🟑**

3) Selection of Controls

a) community controls 

b) hospital controls**🟑**

c) no description

4) Definition of Controls

a) no history of disease (endpoint) 

b) no description of source**🟑**

Comparability

1) Comparability of cases and controls on the basis of the design or analysis

a) study controls for _______________ (Select the most important factor.) **🟑** 

b) study controls for any additional factor  (This criteria could be modified to indicate specific control for a second important factor.)**🟑**

Exposure

1) Ascertainment of exposure

a) secure record (eg surgical records) **🟑**

b) structured interview where blind to case/control status **🟑**

c) interview not blinded to case/control status

d) written self report or medical record only

e) no description

2) Same method of ascertainment for cases and controls

a) yes **🟑**

b) no

3) Non-Response rate

a) same rate for both groups**🟑** 

b) non respondents described

c) rate different and no designation

1. "Mini-mental state". A practical method for grading the cognitive state of patients for the clinician.

**Selection**

1) Is the case definition adequate?

a) yes, with independent validation **🟑** 

b) yes, eg record linkage or based on self reports

c) no description

2) Representativeness of the cases

a) consecutive or obviously representative series of cases 

b) potential for selection biases or not stated**🟑**

3) Selection of Controls

a) community controls **🟑** 

b) hospital controls

c) no description

4) Definition of Controls

a) no history of disease (endpoint) **🟑** 

b) no description of source

Comparability

1) Comparability of cases and controls on the basis of the design or analysis

a) study controls for _______________ (Select the most important factor.) **🟑** 

b) study controls for any additional factor  (This criteria could be modified to indicate specific control for a second important factor.)**🟑**

Exposure

1) Ascertainment of exposure

a) secure record (eg surgical records) 

b) structured interview where blind to case/control status 

c) interview not blinded to case/control status**🟑**

d) written self report or medical record only

e) no description

2) Same method of ascertainment for cases and controls

a) yes **🟑** 

b) no

3) Non-Response rate

a) same rate for both groups **🟑** 

b) non respondents described

c) rate different and no designation

1. Clinical significance of glycemic variability for prediction of cognitive impairment in elderly patients with metabolic syndrome.

**Selection**

1) Is the case definition adequate?

a) yes, with independent validation **🟑** 

b) yes, eg record linkage or based on self reports

c) no description

2) Representativeness of the cases

a) consecutive or obviously representative series of cases 

b) potential for selection biases or not stated**🟑**

3) Selection of Controls

a) community controls 

b) hospital controls

c) no description**🟑**

4) Definition of Controls

a) no history of disease (endpoint) **🟑** 

b) no description of source

Comparability

1) Comparability of cases and controls on the basis of the design or analysis

a) study controls for _______________ (Select the most important factor.) **🟑** 

b) study controls for any additional factor  (This criteria could be modified to indicate specific control for a second important factor.)**🟑**

Exposure

1) Ascertainment of exposure

a) secure record (eg surgical records) **🟑** 

b) structured interview where blind to case/control status **🟑** 

c) interview not blinded to case/control status

d) written self report or medical record only

e) no description

2) Same method of ascertainment for cases and controls

a) yes **🟑** 

b) no

3) Non-Response rate

a) same rate for both groups **🟑** 

b) non respondents described

c) rate different and no designation

1. Relationships between daily acute glucose fluctuations and cognitive performance among aged type 2 diabetic patients.

**Selection**

1) Is the case definition adequate?

a) yes, with independent validation **🟑**

b) yes, eg record linkage or based on self reports

c) no description

2) Representativeness of the cases

a) consecutive or obviously representative series of cases 

b) potential for selection biases or not stated**🟑**

3) Selection of Controls

a) community controls 

b) hospital controls**🟑**

c) no description

4) Definition of Controls

a) no history of disease (endpoint) **🟑**

b) no description of source

Comparability

1) Comparability of cases and controls on the basis of the design or analysis

a) study controls for _______________ (Select the most important factor.) **🟑**

b) study controls for any additional factor  (This criteria could be modified to indicate specific control for a second important factor.)**🟑**

Exposure

1) Ascertainment of exposure

a) secure record (eg surgical records) **🟑**

b) structured interview where blind to case/control status **🟑**

c) interview not blinded to case/control status

d) written self report or medical record only

e) no description

2) Same method of ascertainment for cases and controls

a) yes **🟑**

b) no

3) Non-Response rate

a) same rate for both groups **🟑**

b) non respondents described

c) rate different and no designation

1. Relationship between blood glucose fluctuation and mild cognitive impairment in diabetic patients.

**Selection**

1) Is the case definition adequate?

a) yes, with independent validation **🟑**

b) yes, eg record linkage or based on self reports

c) no description

2) Representativeness of the cases

a) consecutive or obviously representative series of cases 

b) potential for selection biases or not stated**🟑**

3) Selection of Controls

a) community controls 

b) hospital controls**🟑**

c) no description

4) Definition of Controls

a) no history of disease (endpoint) **🟑**

b) no description of source

Comparability

1) Comparability of cases and controls on the basis of the design or analysis

a) study controls for _______________ (Select the most important factor.) **🟑**

b) study controls for any additional factor  (This criteria could be modified to indicate specific control for a second important factor.)**🟑**

Exposure

1) Ascertainment of exposure

a) secure record (eg surgical records) **🟑**

b) structured interview where blind to case/control status **🟑**

c) interview not blinded to case/control status

d) written self report or medical record only

e) no description

2) Same method of ascertainment for cases and controls

a) yes 

b) no**🟑**

3) Non-Response rate

a) same rate for both groups **🟑**

b) non respondents described

c) rate different and no designation

1. Association between Visit-to-Visit Glucose Variability and Cognitive Function in Aged Type 2 Diabetic Patients: A Cross-Sectional Study.

**Selection**

1) Is the case definition adequate?

a) yes, with independent validation**🟑** 

b) yes, eg record linkage or based on self reports

c) no description

2) Representativeness of the cases

a) consecutive or obviously representative series of cases 

b) potential for selection biases or not stated**🟑**

3) Selection of Controls

a) community controls 

b) hospital controls**🟑**

c) no description

4) Definition of Controls

a) no history of disease (endpoint) 

b) no description of source**🟑**

Comparability

1) Comparability of cases and controls on the basis of the design or analysis

a) study controls for _______________ (Select the most important factor.) **🟑**

b) study controls for any additional factor  (This criteria could be modified to indicate specific control for a second important factor.)**🟑**

Exposure

1) Ascertainment of exposure

a) secure record (eg surgical records) **🟑**

b) structured interview where blind to case/control status **🟑**

c) interview not blinded to case/control status

d) written self report or medical record only

e) no description

2) Same method of ascertainment for cases and controls

a) yes **🟑**

b) no

3) Non-Response rate

a) same rate for both groups **🟑**

b) non respondents described

c) rate different and no designation
